# Quality variation patterns in reclaimed pedunculated filament backfill solution during storage

**DOI:** 10.3389/fpls.2026.1751109

**Published:** 2026-05-05

**Authors:** Yudong Yin, Xuan Mai, Jiang Li, Dangan Xiong, Guangxiang Yuan, Li Sun, Yafeng Zhu, Chao Xu, Miaomiao Lu, Guokang Chen, Mengfen Liu, Jiaming Zhao, Zheng Yao, Kunpeng Dou, Kuo Huang

**Affiliations:** 1 China Tobacco Jiangsu Industrial Co., Ltd., Nanjing, China; 2 Zhengzhou Tobacco Research Institute of China National Tobacco Corporation (CNTC), Zhengzhou, China; 3 School of Plant Protection, Southwest University, Chongqing, China; 4 Jiangsu Xinyuan Reconstituted Tobacco Co., Ltd., Huai’an, China

**Keywords:** microorganism, quality, reclaimed pedunculated filament backfill solution, storage, variation patterns

## Abstract

Reclaimed backfill solution, a process-derived liquid generated during pedunculated filament treatment in tobacco manufacturing, has potential value for resource recovery and process reuse; however, its storage stability and deterioration mechanisms remain unclear. To clarify the quality variation patterns during storage, this study analyzed the microbial community composition at different storage stages by using amplicon high-throughput sequencing, identified differential metabolites and functional pathways via untargeted metabolomics, and monitored quality indicators over time. The results show that: 1) During storage, the dominant bacteria in the backfill liquid were *Tuberibacillus*, *Bacillus*, and *Lactobacillus*, while the dominant fungi were *Aspergillus*, *Sampaiozyma*, and *Boeremia*. The relative abundances and diversities of these dominant genera were closely associated with the storage stage. 2) Fourteen significantly altered metabolites were identified in the backfill liquid, such as docosahexaenoic acid, dihomo-gamma-linolenic acid, 5-heptadecyl-1,3-benzenediol and 3-oxoadipic acid. Among them, fatty acyl compounds accounted for the largest proportion and were significantly enriched in the unsaturated fatty acid biosynthesis pathway. 3) The deterioration of the backfill liquid occurred on the fifth day of storage. After deterioration, the total alkaloid content and pH of the backfill solution increased, while the total sugar content and acid value decreased, and the viscosity change was not obvious. Overall, storage-associated shifts in microbial community structure were closely linked to metabolite variation and quality deterioration in the reclaimed backfill solution, with day 5 identified as the critical turning point for quality decline. This finding provides a reference for the management and quality control of other reclaimed or process-derived plant-based liquid systems.

## Introduction

The reclaimed pedunculated filament backfill solution is a tobacco extract that is utilised in the precision manufacturing process of tobacco products, with the purpose of filling empty tobacco stems. It has functions such as improving the burning performance of tobacco strands and enhancing the sensory characteristics of cigarettes (Lu et al., 2021; Tian et al., 2024; Shi et al., 2025). Therefore, its quality is a key factor in ensuring the stable quality of reconstituted stems and tobacco products. However, the regenerated tobacco stem filler solution is prone to microbial contamination during storage, which adversely affects its efficacy and the overall quality of tobacco products, posing significant challenges to production. Moreover, as the tobacco industry raises its quality standards, stringent quality control of this filler solution has become imperative. Therefore, conducting in-depth research on the quality degradation patterns of the filler solution during storage and elucidating its deterioration mechanisms holds substantial practical significance for guiding production practices and ensuring product quality.

During the processing phase, complex processes and flexible production schedules may lead to the accumulation and storage of backfill liquid. The stored backfill liquid contains abundant carbon sources, nitrogen sources, and other nutrients that provide favorable conditions for microbial proliferation (Wang et al., 2014; Yang et al., 2018; Xin, 2021; Liu et al., 2024). Metabolic products, such as enzymes, organic acids and alcohols, are secreted by microbial metabolic activities. These can then interact with the backfill liquid, causing its originally stable pH to change (Shen et al., 2024). Additionally, the volatile sulfur compounds and aldehyde-ketone compounds released by the backfill liquid can lead to the accumulation of characteristic putrid odors, resulting in a significant decline in quality (Yan et al., 2008; Liu et al., 2018). Dang et al. (2018) pointed out that changes in microbial community diversity are the main cause of tobacco base material deterioration (Dang et al., 2018). Ning Yong et al. (2019) found that tobacco extracts prepared through multi-strain co-fermentation not only failed to effectively improve cigarette aroma quality and inhibit the release of off-odors, but also produced microbial-derived off-odors due to microbial metabolism (Ning et al., 2019). These studies indicate that microbial metabolic activities closely regulate the quality changes of various tobacco extracts.

However, existing research on changes in the quality of tobacco products during storage has mainly focused on flue-cured tobacco, reconstituted tobacco stems, and reconstituted tobacco leaves (Zhu, 2013; Huang et al., 2016; Peng et al., 2016). The research on the quality changes of reclaimed pedunculated filament backfill solution during storage is still insufficient. This study provides benefits at both the practical and scientific levels. Practically, identifying the onset of deterioration and the associated quality indicators can support storage management, quality control, and resource-efficient reuse of reclaimed process-derived solutions. Scientifically, integrating microbial community profiling with untargeted metabolomics helps elucidate how storage-associated microbial succession is linked to metabolite shifts and quality deterioration, offering an analytical framework that may also be applicable to other reclaimed plant-derived liquid systems.

## Methods

### Materials and sample preparation

The information of the backfill liquid samples used in this study and other cigarette manufacturing materials used in this study is shown in **Table 1**. The GS01 reclaimed pedunculated filament backfill liquid produced was stored at 20°C, with samples taken on days 1, 3, 5, and 7 (The preliminary experiment and enterprise survey results have basically confirmed that the deterioration occurs in the storage of 3–5 days). The backfill liquid sample stored for 0 days was used as a blank control. Systematically analyzed the microbial community diversity, metabolic product composition, and changes in physicochemical parameters at each storage stage. Then, apply the backfill liquid uniformly to the JSN08 substrate at a coating rate of 38%, complete the humidity equilibrium treatment, cut the strands, and manually roll them into cigarettes for sensory evaluation experiments. Each sample was replicated three times.

**Table 1:** Information of backfill liquid and cigarette preparation material.

Material	Variety type
Backfill liquid sample	GS01 reclaimed pedunculated filament backfill liquid
Cigarette preparation materials	Blank stem fibers
	Regenerated tobacco leaf base
	JSN08 Tobacco stem concentrate
	Tobacco dust concentrate
	Coating liquid

The sample numbers used in this study for amplicon sequencing, non-targeted metabolomics sequencing, and quality change analysis are shown in **Table 2**.

**Table 2:** Sample number information of GS01 backfill liquid taken at different storage stages.

Sample information	Sequencing sample name		Quality analysis sample name
	Amplicon sequencing	Non-targeted metabolomics sequencing	
Stored at 20°C for 0 days	CK	CK	CK
Stored at 20°C for 1 days	KZ201	DX201	D1
Stored at 20°C for 3 days	KZ203	DX203	D3
Stored at 20°C for 5 days	KZ205	DX205	D5
Stored at 20°C for 7 days	KZ207	DX207	D7

### Microbiome sequencing

Total genomic DNA from backfill fluid samples with different storage periods was extracted using the E.Z.N.A.^®^ soil DNA kit. For bacteria, specific primers with barcodes were selected (338F: 5’-ACTCCTACGGGAGGCAGCAG-3’, 806R: 5’-GGACTACHVGGGTWTCTAAT-3’) to amplify the V3 ~ V4 variable region of the 16S rRNA gene, while fungi were amplified using the ITS1F–ITS2R primer sequence (Liang et al., 2024). The amplified products were purified using a 2% agarose gel and a DNA gel purification kit, followed by sequencing on the Illumina NextSeq 2000 platform.

### Metabolomic analysis

Add 400 μL of backfill solution sample and an equal volume of acetonitrile-methanol (1:1, v/v) extraction solution to a centrifuge tube. Mix by vortexing for 30 seconds, then subject to low-temperature ultrasonic treatment at 5°C and 40 kHz for 30 minutes, followed by incubation at - 20°C for 30 minutes. The sample is then centrifuged at 4°C at 13,000 g for 15 minutes, the supernatant is removed, and 100 μL of acetonitrile-water (1:1, v/v) is added for redissolution. The sample is subjected to low-temperature ultrasonic extraction for 5 minutes, centrifuged for 5 minutes, and the supernatant is analyzed by instrumentation. Quality control (QC) samples were prepared by mixing equal volumes of all sample metabolites. During instrument analysis, one QC sample was inserted every three analytical samples to assess system reproducibility. After extraction, the original data file acquisited by LC-MS was converted into mzXML format by ProteoWizard software. Peak extraction, peak alignment and retention time correction were respectively performed by XCMS program. The “SVR” method was used to correct the peak area. The peaks with detetion rate lower than 50% in each group of samples were discarded. After that, metabolic identification information was obtained by searching the laboratory’s self-built database, integrated public database, AI database and metDNA. The chromatographic and mass spectrometric conditions and parameters used for analysis are shown in **Table 3**.

**Table 3:** LC-MS/MS analysis conditions and parameters.

Project conditions		Parameter setting
Chromatographic conditions	Sample volume	2 µL
	Column	HSS T3(100 mm × 2.1 mm i.d., 1.8um)
	Column temperature	40°C
	Mobile phase A	95% water + 5% acetonitrile (containing 0.1% formic acid)
	Mobile phase B	47.5% acetonitrile + 47.5% isopropanol + 5% water (containing 0.1% formic acid)
	Separation gradient	0 ~ 3.5 min, mobile phase B increases to 24.5%, flow rate is 0.40 mL/min
		3.5 ~ 5.0 min, mobile phase B increases to 65%, flow rate remains unchanged
		5.0 ~ 5.5 min, mobile phase B increases to 100%, flow rate unchanged
		5.5 ~ 7.4 min, mobile phase B maintained at 100%, flow rate increases to 0.60 mL/min
		7.4 ~ 7.6 min, mobile phase B decreases to 51.5%, flow rate unchanged
		7.64 ~ 7.80 min, mobile phase B decreases to 0%, flow rate decreases to 0.50 mL/min
		7.8 ~ 9.0 min, mobile phase B decreases to 0%, flow rate decreases to 0.40 mL/min
		9 ~ 10 min, mobile phase B maintained at 0%, flow rate unchanged
Mass spectrometry conditions	Signal acquisition mode	Positive/negative ion scanning mode
	Mass scanning range	70 ~ 1050 m/z
	Sheath gas flow rate	50 psi
	Auxiliary gas flow rate	13 psi
	Ion spray voltage	± 3500 V
	Ion transfer tube temperature	325°C
	Normalized collision energy	20 - 40–60 V cyclic collision energy
	Mass spectrometry resolution	Tier 1: 60,000, Tier 2: 7,500
	Data acquisition mode	DDA mode

### Quality index testing

(1) Chemical parameter testing: In accordance with the methods specified in relevant testing standards, the 305D fully automatic chemical analyzer was used to determine the content of routine chemical parameters (water-soluble total sugars and total plant alkaloids) in samples of backfill liquid from each storage period (State Tobacco Monopoly Administration, 2000; State Tobacco Monopoly Administration, 2019). (2) Physical parameter testing: pH and viscosity were measured using relevant instruments, and acid value testing was conducted in accordance with the method specified in YC/T 145.1-2012 “Tobacco flavor - Determination of Acid Value” (State Tobacco Monopoly Administration, 2012). (3) Sensory quality evaluation: The appearance and aroma changes of backfill liquid from different storage periods were observed and smelled, and the sensory characteristics of cigarette products filled with backfill liquid were evaluated according to YC/T 498-2014 “Tobacco in processing - Sensory evaluation methods” (State Tobacco Monopoly Administration, 2014).

### Data processing and statistical analysis

(1) Based on the methods of Zhou et al. (2025) and Liang et al. (2024), high-throughput sequencing data were analyzed and annotated at the species level (confidence threshold of 70%), followed by the calculation of community composition at different taxonomic levels for each sample and analysis of microbial alpha diversity (Liang et al., 2024; Zhou et al., 2025). (2) Metabolomics data were analyzed using the method described by Yao et al. (2025) (Yao et al., 2025). After metabolite identification in the HMDB database, Metlin database, and Meiji’s self-built database, the R software package ropls (Version 1.6.2) was used for differential metabolite screening (FC = 1.1), and metabolic pathway annotation and pathway enrichment analysis were performed using the KEGG database and the Python package scipy.stats. The correlation analysis of dominant microorganisms, differential metabolites and physicochemical indexes was based on Spearman, and the euclidean distance algorithm was used.

## Results

### Analysis of microbial community diversity in backfill solution with different storage periods

(1) Analysis of species diversity in backfill solution: In the bacterial community, the backfill liquid sample stored for 3 days (KZ203) had the lowest OTU count, Chao, Shannon, and Shannoneven indices, indicating that the microbial community structure may undergo significant adjustments during the initial storage period, leading to a decrease in bacterial diversity, richness, and evenness. By day 5, the Chao and Shannon indices of the backfill liquid sample (KZ205) had significantly increased and reached their peak. By day 7 (KZ207), the Shannoneven index and OTU count also reached their maximum values, indicating that bacterial species diversity and richness recovered and further increased during the mid-to-late storage period, with species distribution becoming more uniform, forming a complex and stable community structure. Amplifier-based high-throughput sequencing analysis revealed (**Table 4**) that the microbial community diversity of the reclaimed pedunculated filament backfill solution exhibited a regular succession pattern as storage time increased.

**Table 4:** Statistical table of microbial community diversity in backfill liquid samples.

Species	Sample treatment	Number of OTUs	Coverage rate	Chao index	Shannon index	Shannoneven index
Bacteria	CK	334	0.997973	392.000000	2.127959	0.366186
	KZ201	344	0.998042	391.600000	2.130553	0.364781
	KZ203	323	0.998295	358.077922	2.049622	0.354750
	KZ205	376	0.997627	462.114754	2.264210	0.381849
	KZ207	391	0.998019	441.763889	2.450391	0.410540
Fungi	CK	519	0.996693	599.294118	3.383616	0.541214
	KZ201	531	0.996819	604.188406	3.496829	0.557285
	KZ203	495	0.997575	539.333333	3.557132	0.573309
	KZ205	366	0.998992	383.103448	3.597754	0.609517
	KZ207	361	0.999370	369.260870	3.508620	0.595805

In fungal communities, the number of OTUs and the Chao index in backfill liquid samples reached their highest levels on the first day of storage (KZ201), while the Shannon and Shannoneven indices peaked on the fifth day (KZ205). This indicates that as storage time increases, the high richness of fungal communities in backfill liquid gradually decreases, but species diversity and evenness continue to increase. By day 7, certain fungal groups may dominate, leading to a decrease in community evenness and a reduction in diversity.

**Figure 1:**
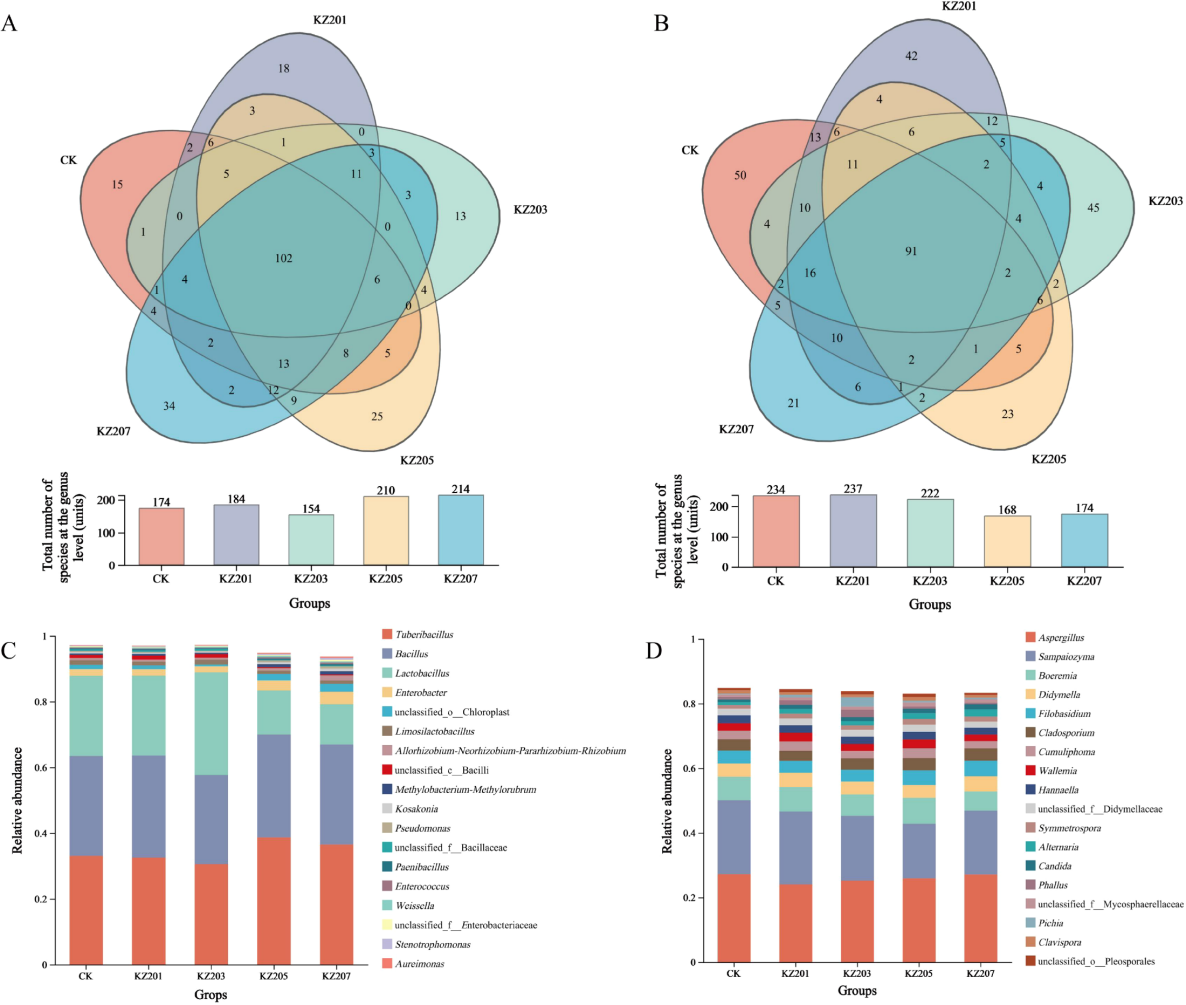
Analysis of species composition in the backfill solution under different storage days. **(A)** Venn diagram of bacterial species composition in the backfill solution during each storage period, **(B)** Venn diagram of fungal species composition in the backfill solution during each storage period; **(C)** Bacterial community composition of backfill solution during each storage period, **(D)** Fungal community composition of backfill solution during each storage period.

(2) Analysis of species composition in backfill solution: The results of the species composition analysis (**Figures 1A, B**) showed that the backfill liquid samples from each storage period contained a total of 102 shared bacterial genera and 91 shared fungal genera. The results of the community composition bar charts (**Figures 1C, D**) indicated the top three genera in terms of abundance remained consistent throughout the backfill liquid storage stages: in bacteria, these were *Tuberibacillus*, *Bacillus*, and *Lactobacillus*; and in fungi, were *Aspergillus*, *Sampaiozyma*, and *Boeremia*. This shows that the core microbial groups exhibit a certain degree of stability during storage.

Additionally, by observing the overall trend of microbial changes in the backfill solution during storage, this study found that on the fifth day, the number of bacterial and fungal genera in the backfill solution underwent significant changes, with a notable increase in the total number of bacterial genera and a significant decrease in the total number of fungal genera (**Figures 1A, B**). Furthermore, fluctuations in bacterial community numbers were more pronounced than those in fungal communities (**Figures 1C, D**). These changes may be key factors in the alteration of backfill solution quality during storage. Among the bacterial communities, the most dramatic changes were observed in the abundance of *Tuberibacillus* and *Lactobacillus*. On day 3, the abundance of *Lactobacillus* increased sharply and temporarily surpassed *Tuberibacillus*, which had maintained an absolute advantage throughout the initial and final stages of storage. On day 5, the abundance of *Lactobacillus* dropped rapidly, while *Tuberibacillus* reached its abundance peak (36.79%). And among fungal communities, the most notable fluctuations were observed between *Sampaiozyma* and *Boeremia*. On day 5, the abundance of *Boeremia* decreased, while the abundance of *Boeremia* reached its maximum value (8.03%); but on day 7, the abundance of *Sampaiozyma* recovered, and *Boeremia* dropped to its lowest abundance value (6.06%).

### Analysis of metabolite composition in backfill solution with different storage periods

**Figure 2:**
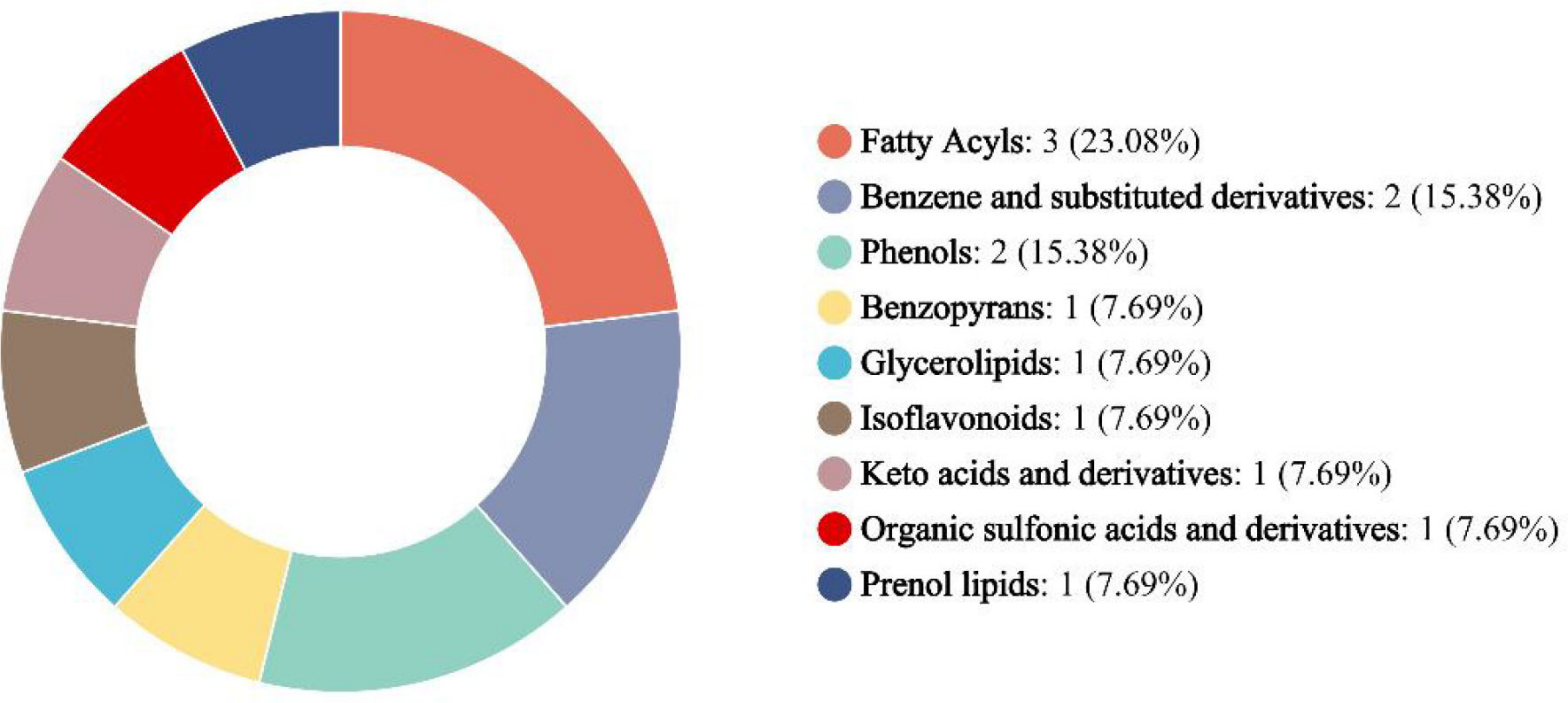
Types of differential metabolites in the backfill solution.

**Figure 3:**
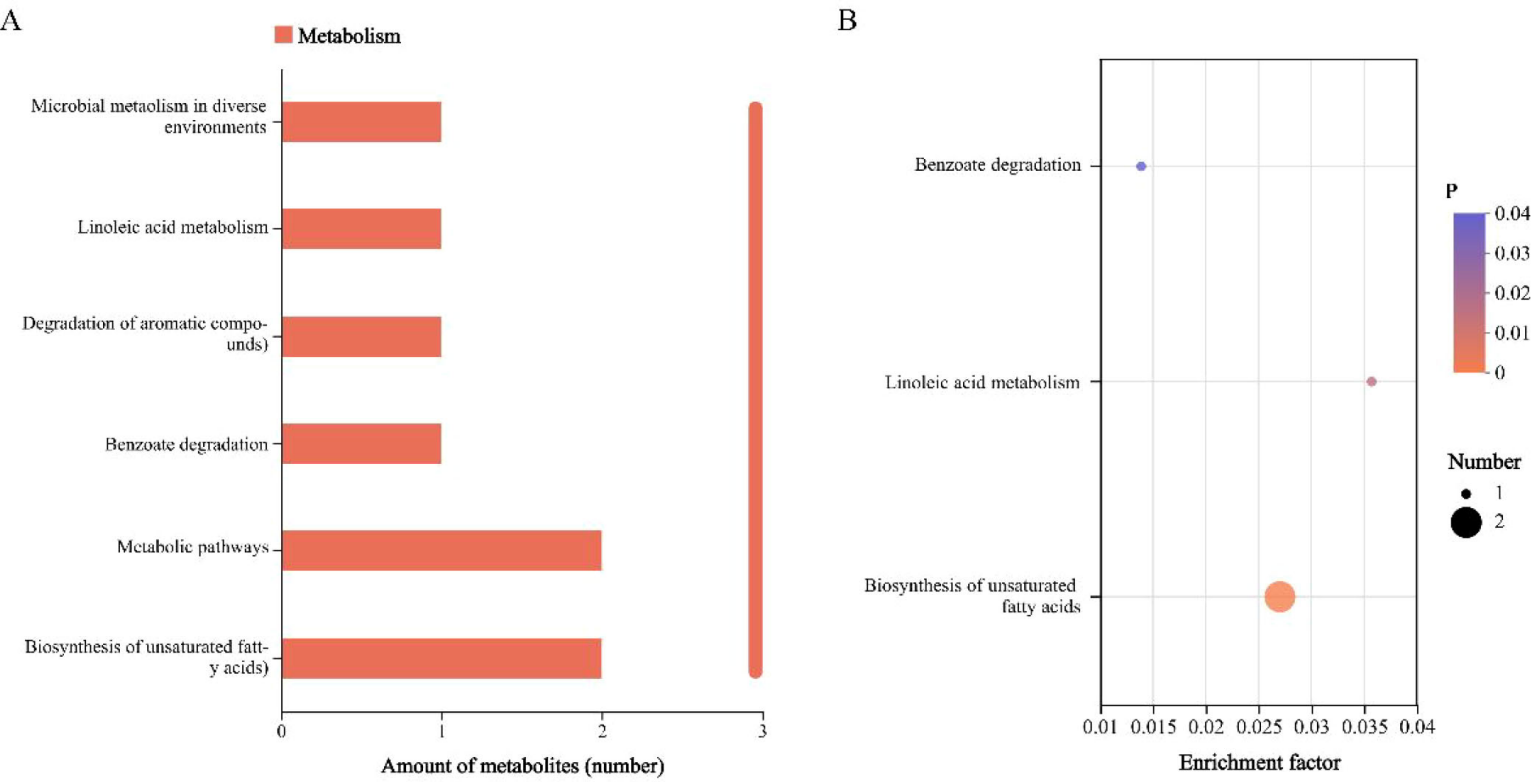
Statistical figures **(A)** and enrichment situations **(B)** of the KEGG pathway of the backfill fluid during each storage period.

(1) Screening of differential metabolites: Using a fold change (FC) of 1.1 as the screening criterion, 14 significantly different metabolites were obtained from the reclaimed pedunculated filament backfill solution of different storage periods. These metabolites include docosahexaenoic acid, dihomo-gamma-linolenic acid, 10Z-nonadecenoic acid, tocainide, dodecylbenzenesulfonic acid, 2-ethylphenol, 5-heptadecyl-1,3-benzenediol, 7-Beta-hydroxy-delta-9-THC, batyl alcohol, shoyuflavone B, 3-oxoadipic acid, 2,5-dihydroxy-1-octadec-9-enoyloxypyrrole-3-sulfonic acid, sterebin B, and prostaglandin D1 alcohol. After annotation by the HMDB database, the aforementioned metabolites can be classified into 9 categories (see **Figure 2**). Among them, fatty acid compounds accounted for the highest proportion (23.08%), and docosahexaenoic acid and 10Z-nonadecenoic acid in this category have special odors; the next were benzene and substituted derivatives and phenols, both accounting for 15.38%, with tocainide having a bitter or aromatic odor, and 2-ethylphenol having an aromatic odor.

(2) Metabolic pathway enrichment analysis: Analysis of the expression levels of 14 differential metabolites revealed that their expression was generally upregulated after storage in backfill liquid, but predominantly showed a downregulation trend during storage (**Table 5**). The period from day 3 to day 7 was identified as the critical phase for significant metabolic changes. Among these, 7-β-hydroxy-δ-9-tetrahydrocannabinol, 8,11,14-eicosatrienoic acid, eicosapentaenoic acid, octadecylglycerol ether, 5-heptadecylresorcinol, 10Z-nonenenoic acid, surfactant D, and 3-oxohexanedioic acid exhibited marked downregulation during days 3 to 5 of storage, while 2-ethylphenol and prostaglandin D1 alcohol showed substantial expression declines between days 5 and 7. The metabolic pathway enrichment results are shown in **Figure 3**. The results indicate that six metabolic pathways were detected in the backfill liquid samples from each storage period, including: microbial metabolism in diverse environments, linoleic acid metabolism, degradation of aromatic compounds, benzoate degradation, metabolic pathways, and biosynthesis of unsaturated fatty acids. Further analysis found that the benzoate degradation, metabolic pathways, and biosynthesis of unsaturated fatty acids pathway were most closely associated with the differential metabolites in the backfill liquid. Among these, the biosynthesis of unsaturated fatty acids pathway not only had the strongest association with the differential metabolites but also involved a greater number of differential metabolites compared to other metabolic pathways, suggesting that the microbial community in the backfill liquid exhibits high activity in such metabolic activities.

**Table 5:** Expression of metabolites differing in backfill fluid during storage.

Metabolite name	Metabolite expression levels				
	CK	KZ201	KZ203	KZ205	KZ207
7-β-hydroxy-δ-9-tetrahydrocannabinol	5.74	6.42	6.43	6.30	6.29
2,5-Dihydroxy-1-octadecyl-9-acyloxy-pyrrole-3-sulfonic acid	4.99	5.60	5.85	5.79	5.74
Tocainide	4.75	5.33	5.36	5.39	5.32
Steviol B	5.20	5.81	5.77	5.77	5.75
2-ethylphenol	5.11	4.81	5.05	5.04	4.65
8,11,14-Tetradecanoic acid	4.54	4.63	5.05	4.83	4.81
Docosahexenoic acid	4.01	4.87	4.45	3.50	4.40
Batyl alcohol	4.26	4.80	4.61	4.39	4.37
5-Heptadecyl-mellodiol	4.60	5.11	4.94	4.62	4.65
10Z-nonenene	4.48	4.96	4.74	4.26	4.42
Surface active agent D	5.15	5.68	5.71	5.59	5.61
3-Oxohexanedioic acid	4.44	4.41	4.50	3.94	3.54
2-hydroxy-3-((5-hydroxy-3-(4-hydroxyphenyl)-4-oxo-4H-benzopyran-7-yl)oxy)succinic acid	4.78	5.30	5.20	5.30	5.25
Prostaglandin D1 alcohol	4.57	4.33	4.92	4.73	4.41

### Analysis of quality changes in backfill solution with different storage periods

(1) Sensory evaluation results: Sensory changes are the direct manifestation of microbial metabolic activities and chemical composition changes in backfilling liquid. The sensory changes in the backfill liquid samples and the cigarettes made from them during each storage period are shown in **Table 6** and **Table 7**. By the third day of storage, the tobacco’s natural aroma in the backfill liquid began to weaken, a slight fermentation odor appeared, and a small amount of sediment formed at the bottom of the bottle. At the same time, the aroma of the cigarettes improved slightly, but the smoke concentration began to deteriorate, and the overall sensory quality remained largely unchanged compared to the first two days of storage. The slight yeast-like sensation in the backfilling liquid during this period may be attributed to the lactic acid produced by the fermentation of Lactobacillus, which has significantly increased in abundance. The sediment at the bottom of the bottle could be high-molecular-weight polymers synthesized by microbial catabolism. By the fifth day of storage, bubbles appeared in the backfill liquid, sediment at the bottom of the bottle increased significantly, the natural tobacco aroma further weakened, and the fermented odor intensified. At this point, the sensory quality of the cigarettes deteriorated significantly, with all evaluation indicators showing marked deterioration, and residual aftertaste remained. It can be seen that the metabolic activity of microorganisms in the backfilling liquid is intensified and the chemical composition changes significantly. By day 7, the deterioration characteristics of the backfill solution such as acidity and fermentation were aggravated, and the cigarette evaluation results were similar to those on day 5. Therefore, this study concludes that the critical point for the backfill liquid’s deterioration is on the fifth day of storage.

**Table 6:** Sensory changes of backfill liquid samples during each storage period.

Sample treatment	Aroma and appearance changes of backfill liquid
CK	Rich sweetness and strong tobacco aroma
D1	Rich sweetness and strong tobacco aroma
D3	Weak tobacco aroma, slight fermentation aroma, sediment at the bottom of the bottle
D5	Bubbles begin to appear, weak tobacco aroma, slight acidity, more sediment at the bottom of the bottle
D7	Bubbles, strong acidity and fermentation aroma, more sediment

**Table 7:** Sensory evaluation of cigarettes made from backfill liquids with different storage times.

Storage days of backfill liquid(d)	Sensory evaluation of cigarettes					
	Aroma quality	Aroma quantity	Offensive odor	Flue gas concentration	Irritant	Aftertaste
0	Moderate	Moderate	Slightly impure	Moderate	Light	None
1	Moderate	Moderate	Slightly impure	Moderate	Light	None
3	Above average	Moderate	Light	Below average	Light	None
5	Poor	Weak	Heavy	Heavy	Heavy	Residual
7	Poor	Weak	Heavy	Heavy	Heavy	Residual

**Figure 4:**
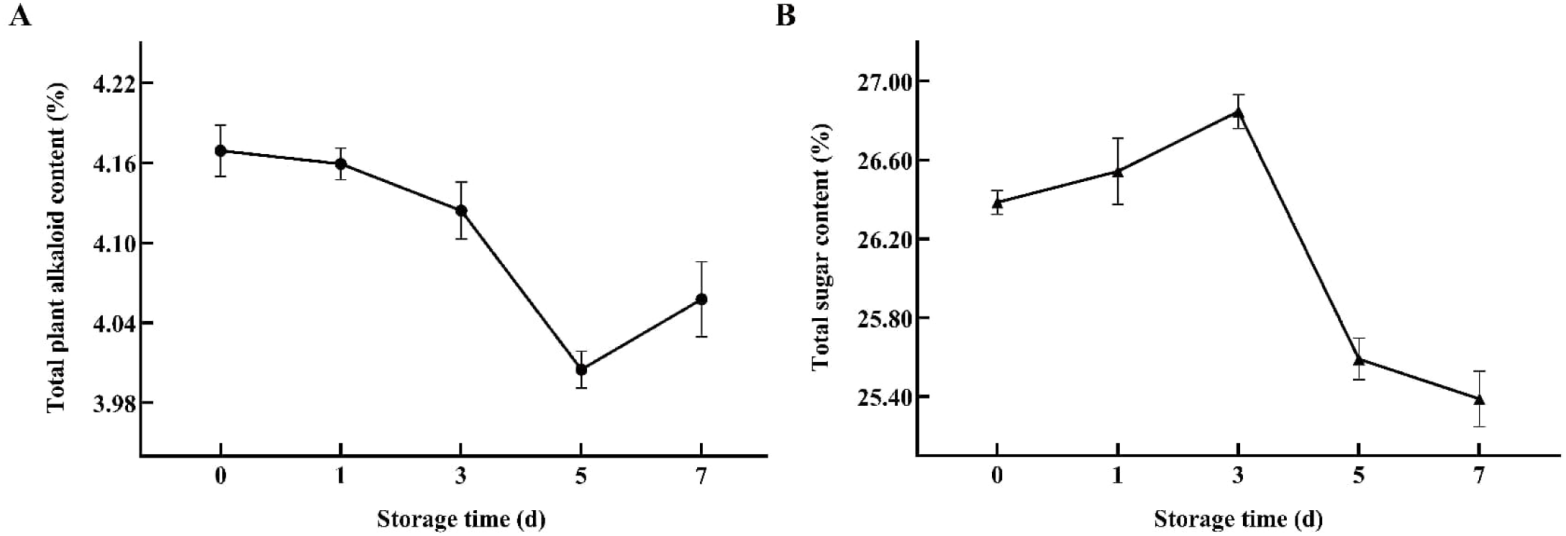
Variations in total plant alkaloids **(A)** and total sugar content **(B)** of backfill liquids with different storage times.

**Figure 5:**
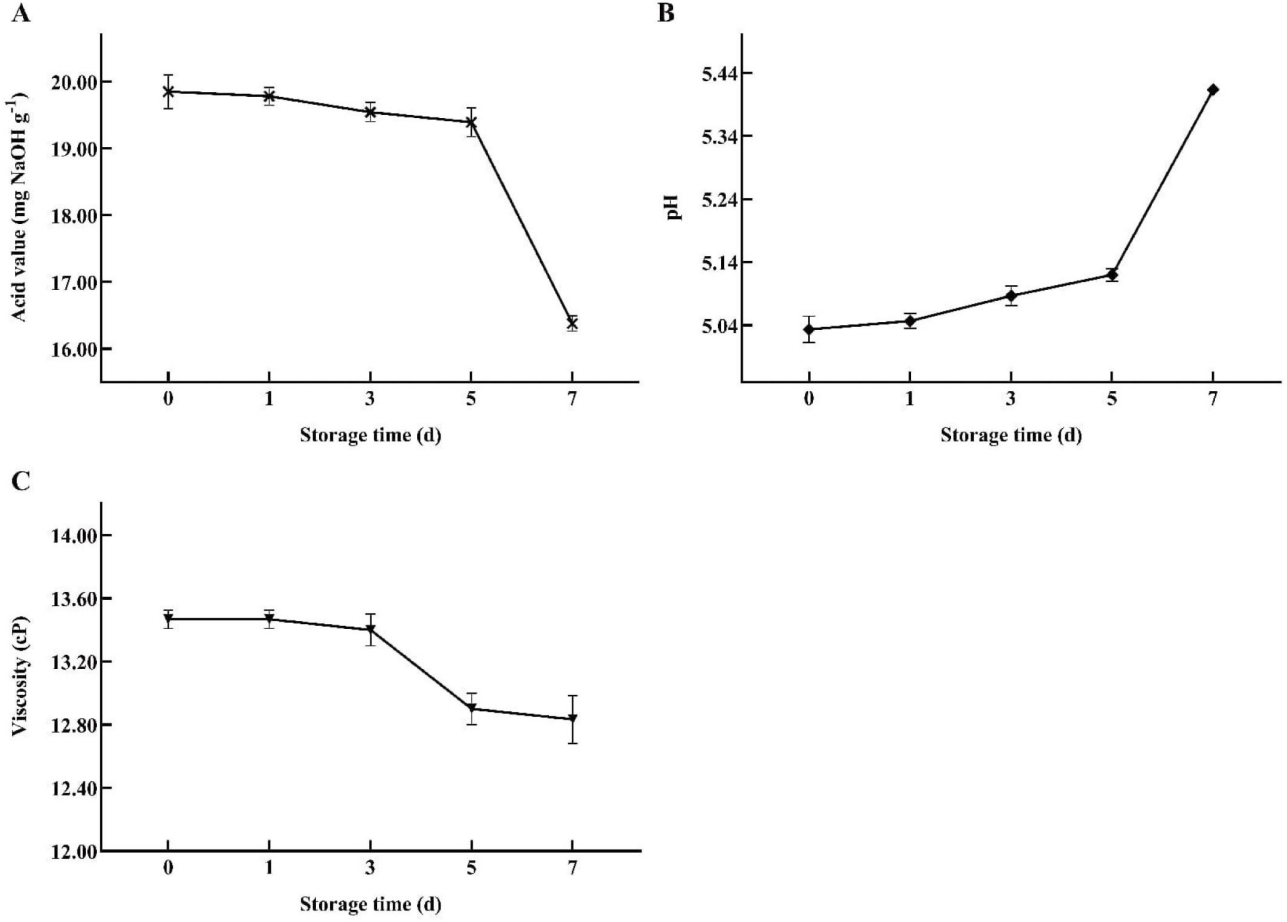
Variations in acid value **(A)**, pH **(B)**, and viscosity **(C)** of backfill liquids with different storage times.

(2) Chemical indicator test results: The results of the determination of total plant alkaloids and total sugar content in the reclaimed pedunculated filament backfill solution are shown in **Figure 4**. The test data showed that, with changes in storage time, the content of total plant alkaloids exhibited a fluctuating pattern of initial decline, followed by a later increase, with the lowest content observed on the fifth day of storage. In contrast, the content of total sugar shows an initial increase followed by a decline in the later stages, reaching its highest value on the third day of storage. Additionally, during the period from the third to the fifth day, the content of both indicators experienced a sharp decline. The results were consistent with the sensory results of the decrease of sweetness and the change of aroma quality and aroma quantity of the backfilling liquid.

(3) Physical indicator test results: According to relevant standards, the acid value, pH, and viscosity of the reclaimed pedunculated filament backfill solution samples with different storage periods were measured (**Figure 5**). It was found that within a 7-day storage period, the viscosity did not show significant fluctuations, with only a brief, slight decrease occurring between days 3 and 5. However, the acid value and pH value fluctuated more obviously, and the change trend of the two was opposite. Specifically, the significant fluctuations in acid value and pH of the backfill liquid primarily occurred between days 5 and 7, during which the acid value sharply decreased while the pH value notably increased. This may be associated with the increased total alkaloid content in the backfill liquid system and the reduction in lactic acid biosynthesis due to a sharp decline in the abundance of *Lactobacillus* species (Cheng et al., 2024). Prior to this period, the changes in acid value and pH were relatively minor. Based on the dynamic change characteristics of the quality index of the backfill liquid during the storage period, the critical point of deterioration of the backfill liquid is determined as the 5th day of storage.

### Analysis of the relevance in backfill solution with different storage periods

**Figure 6:**
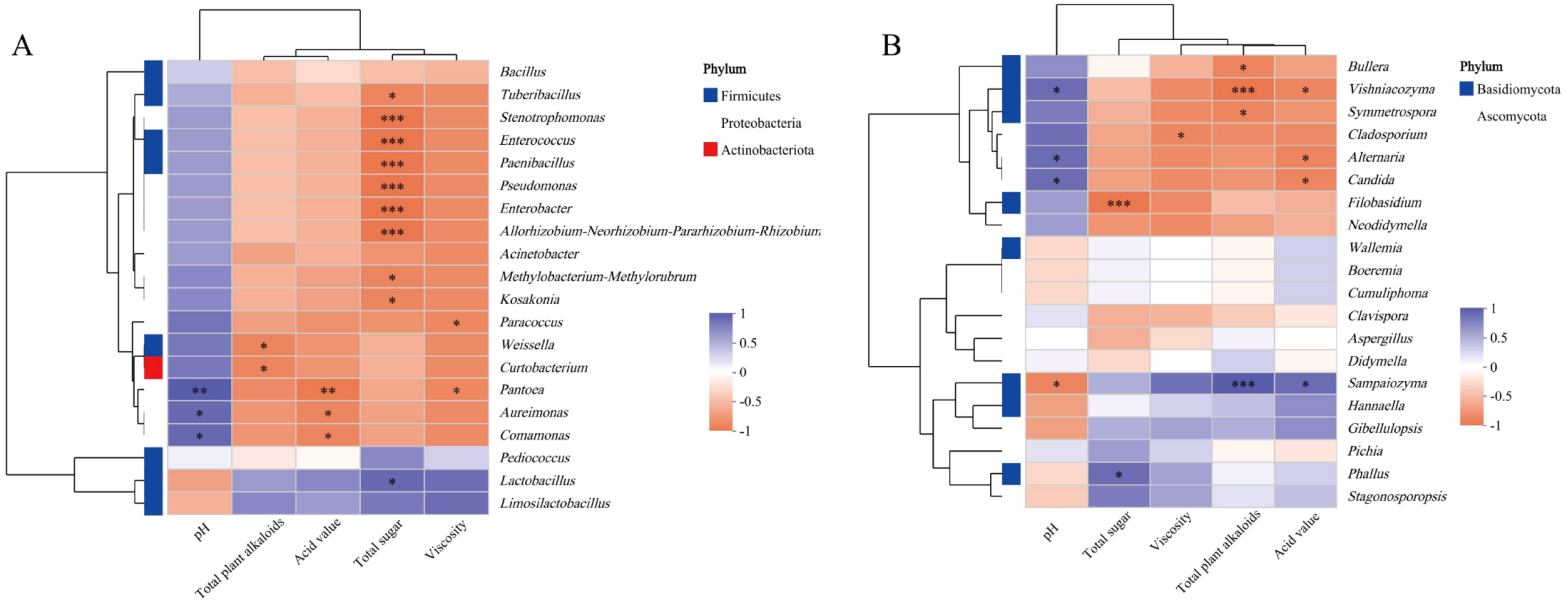
Correlation of bacterial **(A)** and fungal **(B)** species with physicochemical indicators in backfill liquid at the genus level. * represents the magnitude of significance P, * indicates 0.01 <P<0.05; ** indicates 0.001<P<0.01; *** indicates P≤0.001.

A correlation analysis was conducted between the top 20 microbial communities with the highest relative abundance and the physicochemical parameters of the backfill solution. The results revealed that the total sugar content of the backfill solution was most strongly correlated with bacterial community abundance, while the total plant alkaloids content was most strongly correlated with fungal community abundance (**Figure 6**). Specifically, at the genus level, six bacterial genera in the bacterial community - *Stenotrophomonas*, *Enterococcus*, *Paenibacillus*, *Pseudomonas*, etc. - exhibited a highly significant negative correlation with total sugar content (*P* ≤ 0.001). Some strains within these genera possess strong carbohydrate metabolic capabilities and exhibit increased abundance during the mid- and late-storage periods. Therefore, this study speculates that they may contribute to changes in backfill liquid quality by consuming total sugars and thereby promoting the decomposition of the backfill liquid matrix. *Lactobacillus* was significantly positively correlated with total sugar (0.01 < *P* ≤ 0.05) and negatively correlated with pH, indicating that although this genus can synthesize glucan and heteropolysaccharides, its strong acid-producing ability may promote the acidification of the backfill liquid during the later stages of storage. Additionally, analysis revealed that *Pantoea*, *Aureimonas*, and *Comamonas* were positively correlated with pH and negatively correlated with acid value (0.01 < *P* ≤ 0.05), meaning that the decrease in acid value and increase in pH of the backfill liquid are closely related to the reduction in these genera.

In fungal communities, *Bullera*, *Vishniacozyma*, and *Symmetrospora* showed a significant negative correlation with total plant alkaloid content, while *Sampaiozyma* exhibited a strong positive correlation (0.01 < *P* ≤ 0.05). Total plant alkaloids, as characteristic components of the backfill solution, their content changes may affect flavor stability. The aforementioned fungal genera may participate in the quality changes of the backfill solution through metabolic or transformative processes involving total plant alkaloids. Additionally, *Filobasidium* was found to have a highly significant negative correlation with total sugar content, while *Phallus* had a positive correlation with it. The genera *Vishniacozyma*, *Alternaria*, and *Candida* were all negatively correlated with acid value and positively correlated with pH (0.01 < *P* ≤ 0.05).

**Figure 7:**
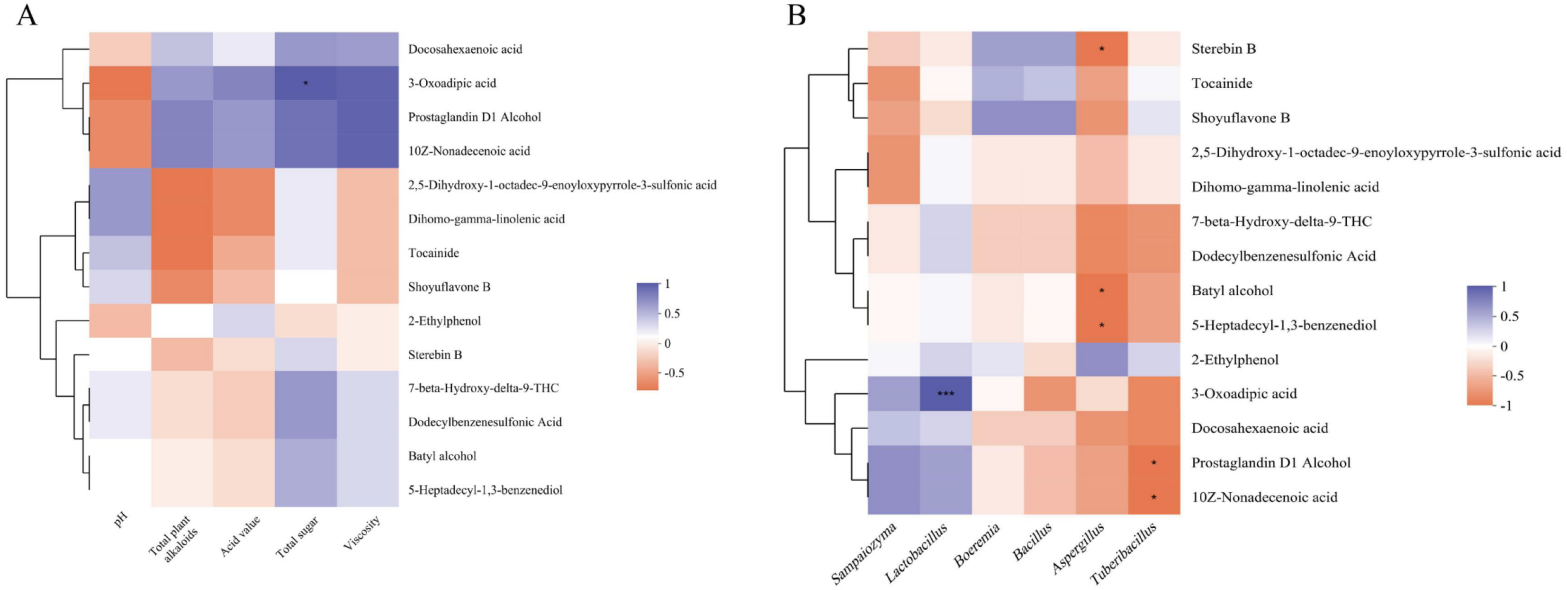
Correlation of physicochemical indicators **(A)** and dominant microbiota **(B)** with differential metabolites in backfill liquid. * represents the magnitude of significance P, * indicates 0.01 <P<0.05; ** indicates 0.001<P<0.01; *** indicates P≤0.001.

The results of the correlation analysis indicate that the differentially expressed metabolites during the storage period of the reclaimed pedunculated filament backfill solution exhibit significant interactive relationships with the microbiome and physicochemical indicators (**Figure 7**). Among the 14 differential metabolites screened in the preliminary experiments, 3-oxohexanedioic acid showed a significant positive correlation with the total sugar content of the backfill solution and the abundance of *Lactobacillus*. The acidic environment generated by *Lactobacillus* during metabolism, along with its competitive inhibitory effect on contaminating bacteria, may facilitate the growth and proliferation of 3-oxohexanedioic acid-producing strains. Consequently, the decline in total sugar levels and the reduction in *Lactobacillus* abundance during the mid-storage phase may alleviate the inhibitory effect on contaminating bacteria, thereby increasing the survival pressure on 3-oxohexanedioic acid-producing strains and subsequently reducing its synthesis. Meanwhile, the continuous metabolism by 3-oxohexanedioic acid-consuming bacteria in the backfill solution further accelerates the consumption of this metabolite. Sterebin B, batyl alcohol, and 5-heptadecyl-1,3-benzenediol were negatively correlated with *Aspergillus*; while prostaglandin D1 alcohol and 10Z-nonadecenoic acid were negatively correlated with *Tuberibacillus* (0.01 < *P* ≤ 0.05). Since glycerol ethers, alkyl resorcinol derivatives, and carbenoid compounds all exhibit certain antibacterial activity, the decline in abundance of *Aspergillus* and *Bacillus thermophilus* during backfill liquor storage may be attributed to these substances. As these compounds were gradually depleted, the abundance of both bacterial genera increased. This study hypothesizes that the initially proliferating *Aspergillus* genus may disrupt the material balance of the backfill liquor through its metabolites, accelerating the release of nutrients and thereby promoting the proliferation of *Bacillus thermophilus* on day 5. The continuously accumulated putrefactive metabolites from these two bacterial genera also reached the putrefaction threshold of the backfill liquor on day 5, ultimately leading to severe deterioration in the quality of both the backfill liquor and the resulting cigarettes.

## Discussion

The core dominant microbial community during the storage period of the reclaimed pedunculated filament backfill solution was composed of the bacterial communities of *Tuberibacillus*, *Bacillus*, and *Lactobacillus*, as well as the fungal communities of *Aspergillus*, *Sampaiozyma*, and *Boeremia*. This dominant status was consistently maintained throughout the study period. *Tuberibacillus*, a thermophilic Gram-positive bacterium, can efficiently degrade volatile organic sulfur compounds and mediate heavy metal detoxification (Kouta et al., 2006; Ding et al., 2019; Han et al., 2022; Zhou et al., 2024). However, no research reports on it have been published in China to date. *Lactobacillus* is commonly found in fermentation systems, and its metabolic activities are closely related to product flavor and human intestinal health (Liang et al., 2023; Liang et al., 2024). Based on the quantitative fluctuation characteristics of both during backfill liquid storage and their correlation with total sugar content, this study hypothesizes that there exists a carbon source competition relationship between *Bacillus thermophilus* and *Lactobacillus*, with differences in their carbon source preferences and utilization rates. Under the high-sugar environment on day 3 of storage, the *Lactobacillus* genus may prioritize the utilization of small-molecule sugars for growth and reproduction. The acidic environment it generates and the bacteriostatic components in the backfill liquid synergistically inhibit the growth of *Bacillus thermophilus*, prompting the genus to enter a dormant state. As small-molecule sugars and bacteriostatic components in the environment are gradually depleted, the *Lactobacillus* genus experiences restricted proliferation due to insufficient carbon sources, thereby alleviating its inhibitory effect on *Bacillus thermophilus*. The latter’s broad-spectrum utilization capacity for carbon sources enables it to proliferate extensively by day 5, releasing more putrefactive substances and accelerating the deterioration of the backfill liquid. Among the other dominant bacterial species, *Bacillus*, *Aspergillus*, and yeasts are widely distributed on tobacco leaves and tobacco products, participating in the process of enhancing aroma and quality (Zhou et al., 2018; Wang et al., 2019; Hu et al., 2023). Zhang et al. (2022) analyzed the microbial community composition of key materials in the remanufactured tobacco production chain and found that *Bacillus*, *Aspergillus*, and *Sampaiozyma* were the dominant genera in many of these materials, with their relative abundances varying depending on the material, thereby influencing the quality of tobacco and its products (Zhang et al., 2022). This conclusion is consistent with the findings of this study.

Through non-targeted metabolomics technology, a total of 14 metabolites with significant differences were screened from the backfill liquid at each storage stage in this study, such as docosahexaenoic acid, dihomo-gamma-linolenic acid, 5-heptadecyl-1,3-benzenediol, 3-oxoadipic acid, etc. Among these substances, those primarily influencing the flavor formation of the backfill liquid from reclaimed pedunculated filament include docosahexaenoic acid, 10Z-nonadecenoic acid, tocainide, 2-ethylphenol, and sterebin B. Literature indicates that docosahexaenoic acid can effectively eliminate off-flavors and enhance flavor characteristics at low concentrations, but excessive accumulation may lead to fishy odors and other negative flavors (Howe et al., 2002; Meadus et al., 2013). Sterebin B, on the other hand, possesses high sweetness characteristics, and the noticeable sweetness in the backfill liquid during the early storage period may be attributed to this substance (Alsafawi and Şahin, 2024). Additionally, some metabolites are closely associated with the dynamic changes in the microbial community of the backfill liquid. For example, alkyl resorcinols can alter the relative abundance of certain microbial communities such as *Fusarium* through their antibacterial effects (Fan et al., 2023); while 3-oxoadipic acid is a key intermediate in the further metabolism of lignin-derived aromatic monomers, and the metabolic activity of lignin-degrading bacterial communities such as *Pseudomonas* in the backfill solution may influence its accumulation levels (Shi, 2020). This study revealed that octadecyl glycerol ether and 5-heptadecyl-mellitol exhibited inhibitory activity against *Aspergillus* species. 10Z-nonenene acid and prostaglandin D1 alcohol significantly reduced the abundance of *Bacillus thermogenes*, while 3-oxohexanedioic acid decreased with the decline in *Lactobacillus* species. These substances interacted with the microbial community, ultimately leading to a marked deterioration in the quality of the backfill liquid by the 5th day of storage.

During the mid-storage period (days 3 - 5), there was a sharp decrease in total plant alkali and total sugar content in the reclaimed pedunculated filament backfill liquid, accompanied by the emergence of off-odors. By the end of the storage period (days 5 - 7), the pH and acid value of the backfill liquid underwent significant changes, with enhanced acidification and fermentation phenomena. Moreover, both stages exhibited a drastic adjustment in the abundance of dominant microbial communities. Therefore, this study defined day 5 of storage as the critical point for the degradation of the backfill liquid. Further analysis revealed that the total sugar content of the backfill solution exhibited a highly significant negative correlation with six bacterial genera in the bacterial community, including *Stenotrophomonas*, *Paenibacillus*, and *Pseudomonas*, and a positive correlation with *Lactobacillus*. The decline in sugar content during the mid-storage period may be attributed to the sharp reduction in *Lactobacillus* abundance during this period, while sugar substrates continue to be consumed by bacteria such as *Paenibacillus* and *Pseudomonas* (Sánchez-Pascuala et al., 2017; Zulema et al., 2018). The total alkaloid content was most strongly associated with the yeast genus in the fungal community. Among them, *Bullera*, *Vishniacozyma*, and *Symmetrospora* showing a negative correlation, while *Sampaiozyma* showed a highly significant positive correlation. This indicates that the decline in total sugar and total alkaloid content on the 5th day of storage is more closely associated with microbial activity. Specifically, the reduction in total sugar content is primarily attributed to metabolic consumption by bacteria such as *Bacillus* and *Pseudomonas*, whereas the decrease in total alkaloid content may be more significantly influenced by the dominant genus, *Saccharomyces pombe*. Wang et al. (2024) pointed out that *Sampaiozyma* may be closely associated with the metabolic processes of various compounds, including amino acids and terpenoids (Wang et al., 2024). Other studies have shown that amino acids and terpenoids are precursor substances for alkaloids (Wang et al., 2023; Zhao et al., 2023). Therefore, this study speculates that *Sampaiozyma* may participate in the synthesis of alkaloids, and the reduction of this genus during the mid-storage period led to a sharp decrease in the total plant alkaloid content of the backfill solution.

Although the studied backfill solution originates from a tobacco-processing system, the significance of this work extends beyond a single industrial context. The present study establishes an integrated strategy combining microbial community analysis, untargeted metabolomics, and physicochemical quality assessment to identify storage-associated deterioration and its potential markers. This framework may be informative for the management and quality control of other reclaimed or process-derived plant-based liquid systems. Nevertheless, the material source and processing characteristics of different industrial liquids may vary, and therefore the specific spoilage markers identified here should be validated before direct extrapolation to other systems.

## Conclusion

This study found that the deterioration of the reconstituted stem silk backfilling solution is closely correlated with storage duration, with day 5 being the critical node for deterioration. As storage time increased, the total bacterial count showed a significant upward trend, while the total fungal count decreased markedly, and the dominant bacterial genera also changed over time. On day 5, the dominant bacteria were *Tuberibacillus*, *Bacillus*, and *Lactobacillus*, and the dominant fungi were *Aspergillus*, *Sampaiozyma*, and *Boeremia*. The degradation pathway of benzoic acid, metabolic pathways, and the synthesis pathway of unsaturated fatty acids showed a high correlation with the differential metabolites in the backfilling solution. Among these, the synthesis pathway of unsaturated fatty acids not only had the strongest association with differential metabolites but also involved more differential metabolites than other metabolic pathways. Fourteen significantly differentially expressed metabolites, including docosahexaenoic acid, 8,11,14-eicosatrienoic acid, 5-hexadecyl-mellitol, and 3-oxohexanedioic acid, changed with storage time. Deterioration not only led to an increase in total alkaloid content and pH value, a decrease in total sugar content and acid value, but also resulted in bubble formation, precipitation, reduced aroma, and subsequent decline in sensory quality.

## Data Availability

The raw sequencing data generated in this study have been submitted to the NCBI Sequence Read Archive (SRA) https://www.ncbi.nlm.nih.gov/, accession number: PRJNA1458996.
